# *Mucuna pruriens* Seed Aqueous Extract Improved Neuroprotective and Acetylcholinesterase Inhibitory Effects Compared with Synthetic L-Dopa

**DOI:** 10.3390/molecules27103131

**Published:** 2022-05-13

**Authors:** Narisa Kamkaen, Chuda Chittasupho, Suwanna Vorarat, Sarin Tadtong, Watoo Phrompittayarat, Siriporn Okonogi, Pakakrong Kwankhao

**Affiliations:** 1 Department of Industrial Pharmacy, School of Pharmacy, Eastern Asia University, Pathum Thani 12110, Thailand; 2 Department of Pharmaceutical Sciences, Faculty of Pharmacy, Chiang Mai University, Chiang Mai 50200, Thailand; siriporn.okonogi@cmu.ac.th; 3 Research Center of Pharmaceutical Nanotechnology, Faculty of Pharmacy, Chiang Mai University, Chiang Mai 50200, Thailand; 4 Department of Pharmaceutical Chemistry, Faculty of Pharmacy, Srinakharinwirot University, Nakhon Nayok 26120, Thailand; suwannav@g.swu.ac.th; 5 Department of Pharmacognosy, Faculty of Pharmacy, Srinakharinwirot University, Nakhon Nayok 26120, Thailand; sarin@g.swu.ac.th; 6 Faculty of Public Health, Naresuan University, Muang, Phitsanulok 65000, Thailand; watoop@nu.ac.th; 7 Chao Phya Abhaibhubejhr Hospital, Ministry of Public Health, Prachin Buri 25000, Thailand; pakakrong2@gmail.com

**Keywords:** *Mucuna pruriens* seed, neuroprotective activity, Parkinson’s disease, anti-acetylcholinesterase activity, L-dopa

## Abstract

L-dopa, a dopaminergic agonist, is the gold standard for the treatment of Parkinson’s disease. However, due to the long-term toxicity and adverse effects of using L-dopa as the first-line therapy for Parkinson’s disease, a search for alternative medications is an important current challenge. Traditional Ayurvedic medicine has suggested the use of *Mucuna pruriens* Linn. (Fabaceae) as an anti-Parkinson’s agent. The present study aimed to quantify the amount of L-dopa in *M. pruriens* seed extract by HPLC analysis. The cytotoxicity and neuroprotective properties of *M. pruriens* aqueous extract were investigated by two in vitro models including the serum deprivation method and co-administration of hydrogen peroxide assay. The results showed the significant neuroprotective activities of *M. pruriens* seed extracts at a concentration of 10 ng/mL. In addition, the effects of L-dopa and *M. pruriens* seed extract on in vitro acetylcholinesterase activities were studied. *M. pruriens* seed extract demonstrated acetylcholinesterase inhibitory activity, while synthetic L-dopa enhanced the activity of the enzyme. It can be concluded that the administration of *M. pruriens* seed might be effective in protecting the brain against neurodegenerative disorders such as Parkinson’s and Alzheimer’s diseases. *M. prurience* seed extract containing L-dopa has shown less acetylcholinesterase activity stimulation compared with L-dopa, suggesting that the extract might have a superior benefit for use in the treatment of Parkinson’s disease.

## 1. Introduction

Due to the low efficiency and toxicity of current medications for Parkinson’s disease treatment, interest is growing in phytochemicals as a potential treatment option. Phytochemicals with neuroprotective activity target various pathways due to their antioxidant, anti-inflammatory, and antiapoptotic properties [1]. *Mucuna pruriens* Linn. is a leguminous plant growing spontaneously in tropical and subtropical areas worldwide. The seeds have traditionally been used in India as a nerve tonic, and a male virility enhancement [2]. In addition, the pods have anthelmintic activity, and the seeds have anti-inflammatory activity. Powdered seeds possess anti-parkinsonism properties, possibly due to the existence of L-dopa, which is a precursor of the neurotransmitter dopamine. The dopamine content in brain tissue is reduced when the conversion of tyrosine to L-dopa is blocked. L-Dopa can cross the blood–brain barrier and undergoes conversion to dopamine, restoring neurotransmission [3]. Particularly, the hydro-alcoholic extract of *M. pruriens* seeds gave high yields of L-dopa, using ascorbic acid as a protector [4]. Surprisingly, n-propanol extract of *M. pruriens* seeds that contained a small amount of L-dopa, yielded the highest reaction for the growth and survival of dopaminergic culture neurons [2]. The significant neuroprotective activity of n-propanol extracts suggested that a whole extract could be potential for the treatment of Parkinson’s disease [5].

Parkinson’s disease is characterized by signs of major oxidative stress and mitochondrial damage in the pars compacta of the substantia nigra [6]. The previous study suggested that reactive oxygen species (ROS) played an important role in age-related neurodegenerative changes including Parkinson’s disease [7]. Interestingly, the ethyl acetate and methanolic extract of the whole *M. pruriens* plant exhibited high antioxidant and free radical scavenging activities [2]. These in vitro assays indicated that the whole plant extract contained large amounts of phenolic compounds, which may be useful in preventing various oxidative stresses. Furthermore, it has been reported that methanolic extracts of *M. pruriens var.utilis* leaves have numerous biochemical and physiological activities, and contain pharmaceutically valuable compounds [8]. However, the in vitro neuroprotective activity of the *M. pruriens* seed aqueous extract obtained from the traditional extraction method has never been reported.

Although the first-line therapy for Parkinson’s disease is L-dopa, long-term L-dopa use results in the development of significant clinical complications. L-dopa-induced dyskinesia (LID) and abnormal involuntary movements (AIMs) normally occur in a vast majority of Parkinson’s disease patients with repeated administration of L-dopa [9]. AIMs occurring at the head, trunk, and extremities affect the function of daily living, and patients can become debilitated. These side effects are common with 30% incidence after 2 years of L-dopa administration. The incidence increased up to 40% and 90% for 5- and 10-year-treatment, respectively [10]. Parkinson’s patients who early started high doses of levodopa are known to have risk factors for LID [11]. At present, few therapeutic options are available for the treatment of LIDs. Several strategies have been proposed to reduce these side effects including postponing the initiation of L-dopa dosing, adjusting the dose of L-dopa, and combining it with other drugs [12,13]. However, these strategies resulted in a decrease in the efficacy of L-dopa in the treatment of Parkinson’s disease.

The seed of *M. pruriens* has been widely investigated for its pharmacological properties including Parkinson’s disease because it contains significant amounts of L-dopa. The advantages of natural L-dopa in *M. pruriens* seed extract over the synthetic forms have been reported. The natural L-dopa is less toxic [14]. It has a shorter onset of action, but it has a longer therapeutic effect, which could delay the need for combination therapy [14,15,16]. In this study, we quantified the amount of L-dopa, total phenolic content, and total flavonoid contents in freeze-dried *M. pruriens* seed extract obtained from an aqueous extraction method. The cytotoxicity and neuroprotective activities of the freeze-dried extract were investigated in neuronal cells using two models. The effects of L-dopa and *M. pruriens* on acetylcholinesterase enzyme activity were studied.

## 2. Materials and Method

### 2.1. Materials

L-dopa and acetylcholinesterase activity assay kit (Cat# CS0003) were purchased from Sigma-Aldrich (St. Louis, MO, USA). Folin–Ciocalteu phenol reagent and aluminum chloride were obtained from Loba Chemie (Mumbai, India). Quercetin (98% purity) was purchased from Chanjao Longevity Co., Ltd. (Bangkok, Thailand). P19 cell line ATCC CRL-1857 was obtained from American Type Culture Collection, USA. Alpha minimal essential medium (α-MEM), fetal bovine serum (FBS), newborn calf serum (NCS), and antibiotics-antimycotic solution were purchased from Gibco, USA. All *trans*-retinoic acid, cytosine-1-β-D-arabinoside, 1:250 porcine trypsin, poly-L-lysine (MW > 300,000), XTT (2,3-bis(2-methoxy-4-nitro-5-sulphonyl)-2H-tetrazolium-5-carboxanilide sodium), and phenazine methosulfate (PMS) were obtained from Sigma, USA. Dimethylsulfoxide (DMSO) and methanol analytical grade were purchased from Merck, Germany. A total of 96-well plates were purchased from Corning, USA. A 100-mm Bacteriological culture dish was obtained from Hycon, USA.

### 2.2. Plant Sample Collection and Identification

The seeds of *M. pruriens* were purchased from a local market in Kanchanaburi province and kindly provided by *Chao-Phraya Abhaibhubejhr* Hospital *Foundation*. The plant materials were compared with the authentic specimens at the Bangkok Herbarium (BK), Botany Section, Botany and Weed Science Division, Department of Agriculture. The voucher specimen (RSU-MP-KP01) was kept in the Department of Pharmacognosy, College of Pharmacy, Rangsit University for future reference.

### 2.3. Plant Extraction

The seeds of *M. pruriens* (10 kg) were roasted by a roasting drum for 30 min at a temperature of 180 °C. The roasting temperature was measured by a sensor located in the drum. The roasted seeds were crushed to fine powders by passing through a stainless-steel sieve with a nominal mesh aperture of 180 µm. The fine powders were extracted with boiling water at 100 °C. The ratio of fine powder to hot water was 1:7. The water extract was mixed using the heating and stirring method with the heater and homogenizer for 15 min. After filtration with the white cloth, the filtrate was then freeze-dried to remove the solvent for 24 h. Finally, the concentrate crude extract was obtained.

### 2.4. HPLC Analysis of L-Dopa in M. pruriens Extract

Isocratic HPLC analysis was performed to analyze the amount of L-dopa remaining in the extract using Perkin Elmer series 200, USA, equipped with auto-sampler and a UV–Vis detector. The injection volume was 20 μL for all samples. The mobile phase for elution was 0.1 M KH_2_PO_4_ (pH 2.5). Samples were eluted on ACE-129-2546 (250 mm × 4.6 mm) C_18_ column with a flow rate of 1.0 mL/min. L-dopa peak was integrated at the wavelength of 283 nm. A stock solution of L-dopa was prepared to obtain L-dopa concentration of 1 mg/mL. The stock solution was diluted to 10, 20, 30, 40, and 50 μg/mL in 0.1 N HCl. *M. pruriens* aqueous extract was weighed and dissolved in 0.1 N HCl. Samples were then filtered through Whatman filter paper and 0.45 μm membrane filter.

### 2.5. Quantitative Analysis of Total Phenolic Content

Total phenolic content in *M. pruriens* seed extract was determined by the Folin–Ciocalteu reaction. Gallic acid solution (3.9–125 µg/mL) and *M. pruriens* seed extract solution (2 mg/mL) were mixed with 10% *v/v* of Folin–Ciocalteu phenol reagent (100 µL) for 1 min. After 4 min of incubation, 7.5% *w/v* Na_2_CO_3_ solution (50 µL) was added, and the mixture was further incubated in the dark at room temperature for 2 h. The absorbance was read using a UV–visible spectrophotometer (Spectramax M3, Thermo Scientific, Waltham, MA, USA) at a wavelength of 765 nm. Total phenolic contents were calculated from the gallic acid standard curve. Data were expressed as mg/g gallic acid equivalents (GAE) of dry crude extract.

### 2.6. Quantitative Analysis of Total Flavonoid Content

The total flavonoid content in *M. pruriens* seed extract was determined by the aluminum chloride colorimetric method. Quercetin solution (3.9–500 µg/mL) and *M. pruriens* seed extract solution (2 mg/mL, 100 µL/well) were mixed with 5% NaNO_2_ (30 µL) and incubated for 5 min. Aluminum chloride (2% *w/v*, 50 µL) was added and incubated for 6 min followed by 10 min of incubation with 1 N NaOH (50 µL). The absorbance was measured at a wavelength of 510 nm with a UV–Vis spectrophotometer (Spectramax M3, Thermo Scientific, Waltham, MA, USA). Total flavonoid contents were calculated from quercetin and were expressed as mg/g quercetin equivalents (QE) of dry crude extract.

### 2.7. Cell Culture

P19 cells (ATCC CRL-1857) were grown in alpha minimal essential medium (α-MEM) supplemented with 7.5% newborn calf serum (NCS), 2.5% fetal bovine serum (FBS), and 1% antibiotics-antimycotic solution in a 5% CO_2_ humidified atmosphere, at 37 °C Cells in monolayer cultures were maintained in exponential growth by subculturing every 2 days [17].

### 2.8. Differentiation of P19 Cells into P19-Derived Neurons

Exponentially grown cultures were trypsinized and dissociated into single cells. P19 cells (2 × 10^6^ cells/mL) were then suspended in 10 mL α-MEM supplemented with 5% FBS, 1% antibiotics-antimycotic solution and 0.5 μM all-*trans*-retinoic acid (RA) and seeded onto a 100-mm bacteriological culture dish. The cells formed large aggregates in suspension. After 4 days of RA treatment, aggregates were dissociated by 5-mL glass measuring pipette, re-plated on poly-L-lysine-pre-coated multi-well plates (multi-well plates were coated with 50 μg/mL poly-L-lysine dissolved in PBS for overnight and sterile under UV light for 30 min) at 7 × 10^4^ cells/mL (150 μL/well in 96-well plate), in α-MEM supplemented with 10% FBS, and 1% antibiotics-antimycotic solution and incubated for 24 h. Cytosine-1-β-D-arabinoside or Ara-C (10 μM) was added at day 1 after plating and the medium was changed every 2–3 days. The differentiated neuronal cells, P19-derived neurons, were used after day 14 of the differentiation process [18,19,20].

### 2.9. Neuronal Cell Viability Assay

The cytotoxicity assay was carried out on P19-derived neurons cultured in a 96-well plate. After 14 days of differentiation process, the α-MEM supplemented with 10% FBS, 10 μM Ara-C, and 1% antibiotics-antimycotic solution (P19SM) was removed, and DMSO solutions of the sample, diluted with the P19SM were added to give the concentrations of 10,000, 1000, 100, 10, and 1 ng/mL [20,21,22,23,24]. The 0.5% *v/v* DMSO in P19SM was used as control. The cells were incubated for 18 h at 37 °C. Then 150 μL of the medium was removed, and 50 μL of XTT solution (1 mg/mL XTT in 60 °C α-MEM + 25 μM PMS) was added. After incubation at 37 °C for 4 h, 100 μL of PBS (phosphate buffer saline solution) pH 7.4 was added. The OD value was determined on a microplate reader at 450 nm. The data were expressed as the average % cell viability ± SE (*n* = 3, each *n* was run in triplicate). The samples that enhanced survival of cultured neurons more than control (0.5% *v/v* DMSO in the medium) will be further investigated for their neuroprotective ability.

### 2.10. Neuroprotective Assay 

The assays were carried out on P19-derived neurons cultured in a 96-well plate and performed for three independent experiments each experiment was run in triplicate [21,22,23,24,25,26].

#### 2.10.1. Serum Deprivation Method

The DMSO solution of the extracts diluted with the α-MEM supplemented with 1% antibiotics–antimycotic solution and 10 μM Ara-C without FBS were added to give the final concentration of the extract at concentration that enhanced survival of cultured neurons more than control. The 0.5% *v/v* DMSO in the completed medium (P19SM, α-MEM supplemented with 1% antibiotics–antimycotic solution and 10 μM Ara-C with 10% *v/v* FBS) was used as control. The 0.5% *v/v* DMSO in α-MEM supplemented with 10 μM Ara-C, and 1% antibiotics–antimycotic solution without FBS was used to make oxidative stress condition. The cells were incubated for 18 h at 37 °C. Cell viability was assayed by XTT reduction method. The data were expressed as the average % cell viability ± SE (*n* = 3) [10,11,12,13,14]. Quercetin at concentration of 1 nM was used as positive control [26].

#### 2.10.2. Co-Administration of H_2_O_2_ Assay

The DMSO solution of the extracts diluted with P19SM plus 10 μM Ara-C was added to give the final concentration of the extract at a concentration that enhanced survival of cultured neurons more than control. The 0.5% *v/v* DMSO in P19SM was used as control. The 5 mM H_2_O_2_ in P19SM plus 10 μM Ara-C was used to make oxidative stress condition. The 5 mM H_2_O_2_ and the extracts in P19SM plus 10 μM Ara-C were added together for co-administration assay. The cells were incubated for 18 h at 37 °C. Cell viability was assayed by XTT reduction method. The data were expressed as the average % cell viability ± SE (*n* = 3) [9]. Quercetin at concentration of 1 nM was used as positive control [26].

### 2.11. Acetylcholinesterase (AChE) Activity Assay

The effects of L-dopa and *M. pruriens* seed extract on the acetylcholinesterase activity were measured using a colorimetric assay based on an Ellman method. Briefly, samples (50 µL) were mixed with acetylcholinesterase enzyme (50 µL) in a 96-well plate. The assay buffer (50 µL) was used as a blank. The reaction was initiated by the addition of 1x substrate mix solution to the sample and blank wells. The hydrolysis of this substrate was immediately monitored by measuring the absorbance at a maximum wavelength of 412 nm in kinetic mode at 1 min-interval for 20 min. The formation of yellow 5-thio-2-nitrobenzoate anion was detected as the result of the reaction of DTNB with thiocholine, released by the enzymatic hydrolysis of acetylthiocholine iodide. The enzymatic activity was calculated as a percentage of the velocity of the reaction with and without the extract. The velocity of the reaction was determined by constructing a kinetic curve between the absorbance and the incubation time. The slope was in units of O.D./min. The absorbance was calculated from the following equation.

A=ε×I×c

where A is the absorbance (in O.D), ε is the extinction coefficient (13,600 L/molxcm), I is the path length of a 96-well plate, and c is the AChE activity in units (µmole.min).

To calculate the enzymatic activity (units/liter), the following equation was used.

$$AChE\;activity=\frac{Slope\;\left(\frac{OD}{\min}\right)\times 10^{-4}\left(liter\right)\times 10^{6}\left(µ\frac{mole}{mole}\right)}{13,600\;\left(\frac{liter}{mole}\times cm\right)\times 0.3\times 5\times 10^{-5}\;\left(liter\right)}$$

### 2.12. Statistical Analysis

The average viability of the neurons was statistically analyzed by one-way ANOVA and further analyzed by Fisher’s LSD to compare the statistical significance between the control or oxidative stress conditions and experimental groups. Differences were considered significant only when the *p*-value was less than 0.05.

## 3. Results

### 3.1. Yield of M. pruriens Aqueous Extract and L-Dopa Content in the Extract

The yield of crude aqueous extract of *M. pruriens* seeds was 15.02%. L-dopa content in the crude aqueous extract analyzed by HPLC analysis was 7.05 + 0.02%.

### 3.2. Total Phenolic and Total Flavonoid Content in M. pruriens Aqueous Extract

The gallic acid standard curve equation was expressed as y = 0.0173x + 0.1011, r^2^ = 0.999. The average total phenolic content in the extract was 105.59 ± 0.84 mg gallic acid equivalent (GAE)/g crude dry extract. The total flavonoid content in *M. pruriens* seed extract determined by the aluminum chloride colorimetric method was expressed in quercetin equivalent amounts. The quercetin standard curve equation was y = 0.0013x + 0.0481, r^2^ = 0.9977. The average total flavonoid content in the extract was 80.74 ± 0.51 mg quercetin equivalent (QE)/g crude dry extract. 

### 3.3. Cell Viability Assay

**Figure 1:**
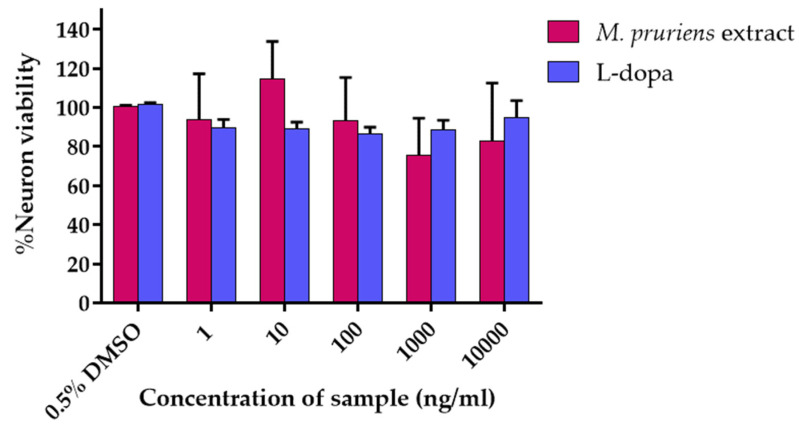
P19-derived neurons cell viability in various concentration (1–10,000 ng/mL) of *Mucuna pruriens* seed aqueous extract and L-dopa.

P19-derived neurons culture was treated with an increasing concentration of *M. pruriens* seed aqueous extract and L-Dopa (0–10 µg/mL). The neuronal viability was determined by XTT assay. The results were expressed as % cell viability. The effective concentration of *M. pruriens* seed aqueous extract at 10 ng/mL (% neuron viability = 114.48 ± 19.25%) that enhanced the survival of cultured neurons more than control (% neuron viability of the control = 100.47 ± 0.67%) were further investigated for the neuroprotective ability (Figure 1). Interestingly, L-dopa showed no enhancing cell viability effect on P19-derived neurons, suggesting that L-dopa was not responsible for the neuroprotective ability of *M. pruriens*. Therefore, only the extract was further investigated for its neuroprotective ability.

### 3.4. Serum Deprivation Method

**Figure 2:**
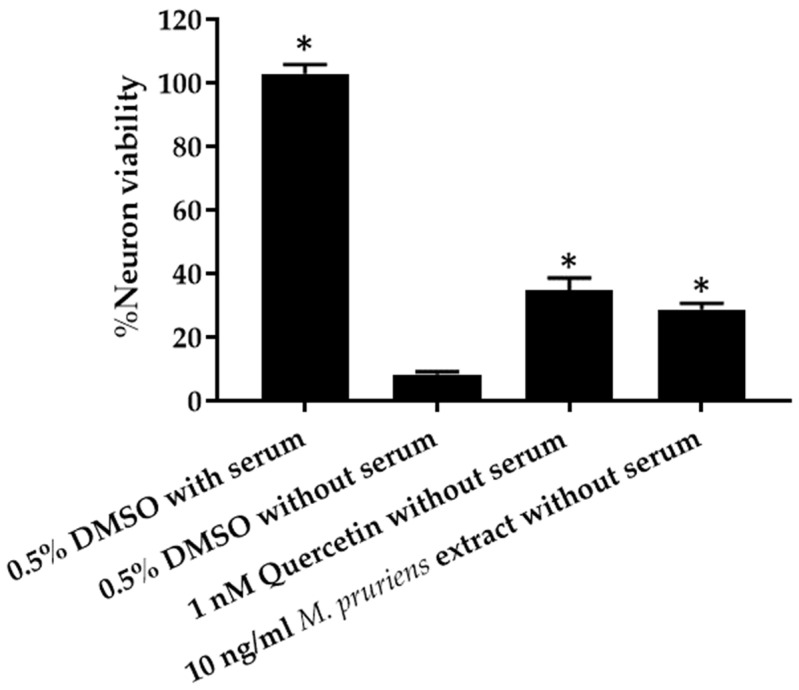
P19-derived neurons cell culture viability in 10 ng/mL *Mucuna pruriens* seed aqueous extract compared with the positive control (1 nM quercetin) and toxic condition (0.5% *v/v* DMSO in α-MEM without serum). * *p* < 0.05 compare with toxic condition (0.5% *v/v* DMSO in α-MEM without serum).

P19-derived neurons culture was treated with 10 ng/mL of *M. pruriens* seed aqueous extract compared with toxic condition (0.5% *v/v* DMSO in α-MEM without serum) and positive control (1 nM quercetin). Cell viability was assayed by XTT reduction method. The results show significant cell viability in 10 ng/mL Mucuna (28.73 ± 1.90%) compared with the toxic condition (8.37 ± 0.73%) (Figure 2). The % neuron viability, when treated with 1 nM quercetin (positive control), was 34.73 ± 3.93%. No significant difference was found between the % neuron viability when treated with 10 ng/mL Mucuna and 1 nM quercetin.

### 3.5. Co-Administration of H_2_O_2_ Assay

**Figure 3:**
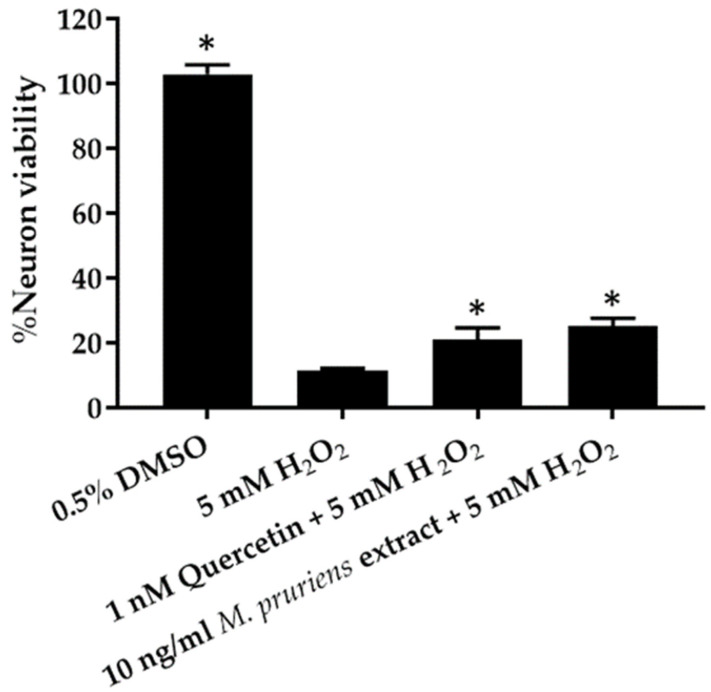
P19-derived neurons cell culture viability in 10 ng/mL *M. pruriens* seed aqueous extract co-treated with 5 mM hydrogen peroxide compared with the positive control (1 nM quercetin co-treated with 5 mM hydrogen peroxide) and toxic condition (5 mM hydrogen peroxide). * *p* < 0.05 compared with toxic condition (5 mM hydrogen peroxide).

P19-derived neurons culture was co-treated with 10 ng/mL of *M. pruriens* seed aqueous extract and 5 mM H_2_O_2_. The cell viability was compared with the toxic condition (5 mM H_2_O_2_). The cell viability was assayed by XTT reduction method. The results showed significant cell viability in 10 ng/mL *M. pruriens* seed extract co-treated with 5 mM H_2_O_2_ (25.01 ± 2.66%), compared with the toxic condition (11.61 ± 0.50%) (Figure 3). The % neuron viability when co-treated with 1 nM quercetin (positive control) and 5 mM H_2_O_2_ was 20.96 ± 3.67%. No significant difference was found between the % neuron viability when treated with 10 ng/mL *M. pruriens* seed extract and 1 nM quercetin.

### 3.6. Effects of L-Dopa and M. pruriens Seed Aqueous Extract on the Acetylcholinesterase Enzyme Activity

**Figure 4:**
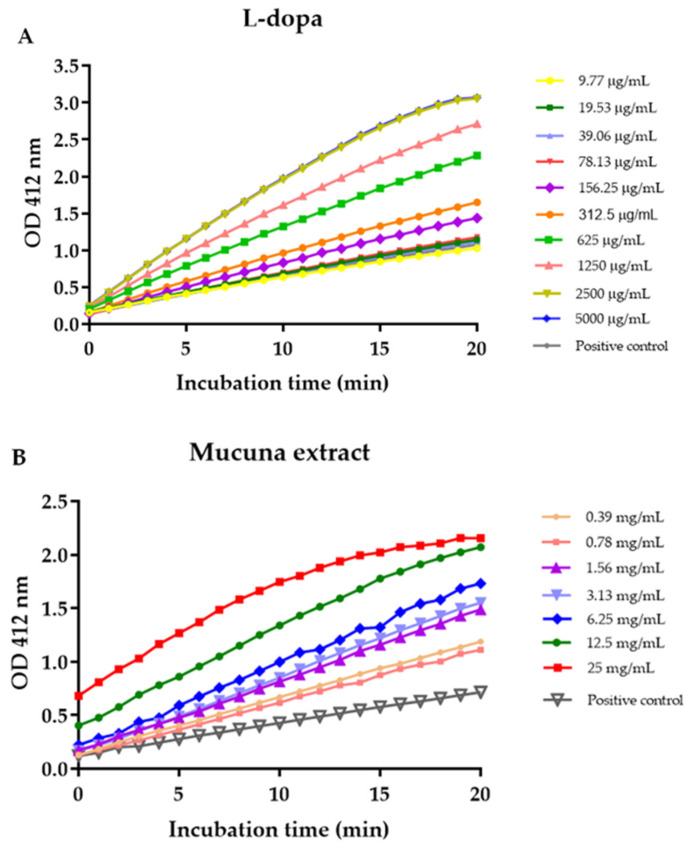
Plots of average O.D. readings subtracted from the mean blank (no enzyme) of each time point from its respective mean sample time points vs. time in the presence of (**A**) L-dopa and (**B**) *M. pruriens* seed extract.

**Figure 5:**
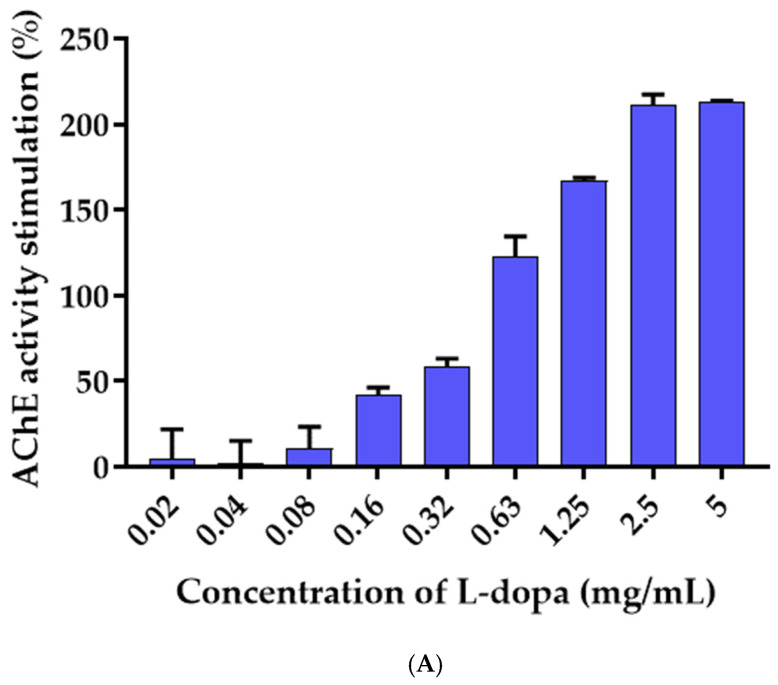

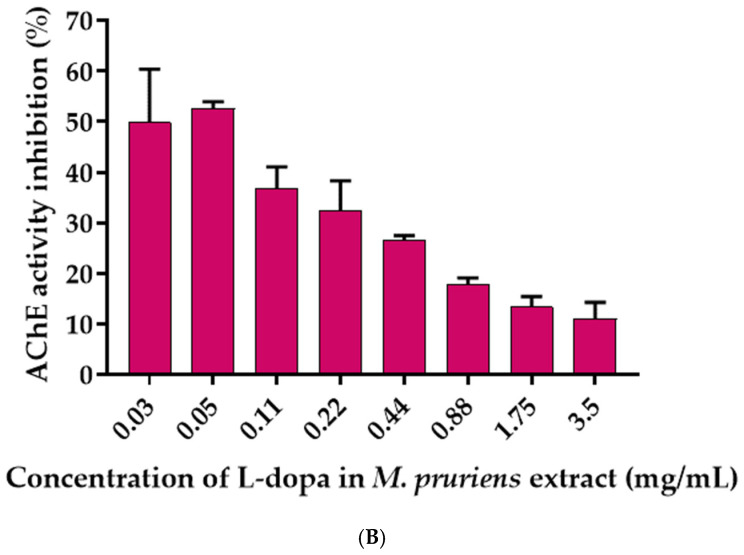
Acetylcholinesterase activity in the presence of (**A**) synthetic L-dopa and (**B**) *M. pruriens* seed aqueous extract.

The effect of L-dopa and *M. pruriens* seed extract on acetylcholinesterase activity was investigated by the Ellman method, in which thiocholine produced by AChE reacted with 5, 5′-dithiobis(2-nitrobenzoic acid) to form a colorimetric product. The enzyme activity of the control without reaction with samples was 2.29 Units/L. One unit of AchE is the amount of enzyme that catalyzes the production of 1.0 µmole of tricholine per minute at pH 8.0 at room temperature. L-dopa was found to increase the activity of the acetylcholinesterase enzyme in a dose-dependent manner, whereas *M. pruriens* seed extract showed inhibitory activity against the acetylcholinesterase enzyme. Figure 4 demonstrated a kinetic curve plotted between the absorbance of the Ellman reaction product and time. The slopes of each plot were further used to calculate the AChE activity. The AChE activity increased with increasing concentrations of L-dopa (Figure 5). At the highest concentration of L-dopa (5 mg/mL), the AChE activity was activated up to 213%. In contrast, M. pruriens seed extract showed enzymatic inhibitory activity. The AChE inhibitory activity of *M. pruriens* seed extract was found to be 52.61% at an L-dopa equivalent concentration of 0.05 mg/mL. However, at the higher dose, the enzyme inhibitory effect was considerably lower to 11.02% at an L-dopa equivalent concentration of 3.5 mg/mL. This result was probably due to a higher concentration of L-dopa present in the extract.

## 4. Discussion

Parkinson’s disease is a progressive neurodegenerative disorder resulting from the damaged dopaminergic neurons in the substantia nigra. The disease recently has no proven neuroprotective or neurorestorative therapy [27]. However, protecting neurons from premature cell death might be a promising alternative to managing this disease. Research directions include the investigation into animal models of the disease and the potential usefulness of gene therapy, stem cell transplant, neuroprotective agents, and herbals drugs.

The present study investigated the neuroprotective activities in P19-derived neurons using two models including the serum deprivation method and co-administration of hydrogen peroxide assay. Remarkably, the significant neuroprotective activities of standardized aqueous extracts of *M. pruriens* seed (L-dopa 7.05 ± 0.02 mg/100 g) were observed. The total phenolic compounds and total flavonoid content in *M. pruriens* seed aqueous extract were 106 GAE mg/g crude extract and 84 QE mg/g crude extract, respectively. Shin et al. have reported that L-dopa and pramipexol (a dopamine agonist) had comparable neuroprotective properties in 1-methyl-4-phenyl-1,2,3,6-tetrahydropyridine (MPTP)-treated Parkinson’s disease animal models through modulation of cell survival and apoptotic pathway [28]. The total phenolic compounds have been shown to exert neuroprotective effects against hydrogen peroxide-induced oxidative damage by blocking reactive oxygen species production and improving mitochondrial function [29]. Flavonoids have demonstrated neuroprotective effect via the inhibition of cholinesterase enzymes including acetylcholinesterase (AChE), butyrylcholinesterase (BChE), and β-secretase (BACE1) [30]. Therefore, our findings suggest that phenolic compounds and flavonoids in *M. pruriens* seed extract might play an important role in the neuroprotective effect against oxidative damage.

Regarding the serum deprivation method, the results demonstrated significant cell viability in 10 ng/mL *M. pruriens* extract (28.73 ± 1.90%) compared with the toxic condition (8.37 ± 0.73%). In addition, the results of the co-administration of hydrogen peroxide assay showed significant cell viability in 10 ng/mL *M. pruriens* extract (25.01 ± 2.66%) compared with the toxic condition (11.61 ± 0.50%). Our results agree with those of some previous studies. The evaluation of the anti-neuroinflammatory effects of *M. pruriens* extract was performed in murine microglia BV-2 cells. [16] *M. pruriens* extract was evaluated for neuroprotective effects in human neuroblastoma SH-SY5Y cells. The study showed that *M. pruriens* extract (at 1 μg/mL and 10 ng/mL) significantly reduced 6-hydroxydopamine-induced cytotoxicity and increased cell viability by 73.1 and 75.1%, respectively. The examination of ethanolic extract of *M. pruriens* on the level of nitric oxide in paraquat-induced Parkinson’s disease mouse model and its subsequent contribution to lipid peroxidation results demonstrated that *M. pruriens* protects the dopaminergic neurons from the NO injury in substantia nigra [31]. The investigation of *M. pruriens* ethanolic seed extract in the Parkinsonian mouse model was performed [32]. A significant reduction in the activity of tyrosine hydroxylase positive neurons was observed in the substantia nigra region of the brain, after treatment with 1-methyl-4-phenyl-1,2,3,6-tetrahydropyridine.

Although the gold standard treatment of Parkinson’s disease is L-dopa dopamine precursor, long-term L-dopa administration resulted in the development of serious side effects, including dyskinesia and the development of fluctuations in motor response. L-dopa-induced dyskinesia in Parkinson’s disease patients significantly contributes to altered cholinergic signaling. Importantly, many studies showed that cholinergic receptor drugs including nicotine resulted in significant declines in L-dopa-induced dyskinesia [33,34,35]. Keeping the dose of L-dopa below 400 mg per day in early Parkinson’s disease has been shown to reduce the risk of dyskinesia induction [36]. In this study, we showed that *M. pruriens* extract containing 7% natural L-dopa decreased the acetylcholine esterase stimulation compared with control. In contrast, the synthetic L-dopa significantly stimulated acetylcholinesterase activity. The results suggested that *M. pruriens* seed aqueous extract was probably able to modulate the cholinergic system and reduce L-dopa-induced dyskinesia and movement disorders resulting from L-dopa treatment. The present study demonstrated the neuroprotective activity of *M. pruriens* seed extract which may imply the therapeutic effects in various nervous system disorders including Parkinson’s disease. Different mechanisms could underline this protection. Since *M. pruriens* seed extract showed neuroprotective activity against hydrogen peroxide-induced neurotoxicity, its proposed antioxidative therapeutics can be used for the protection of neuron injury induced by oxidative stress [37]. Serum deprivation-induced cell death is recognized as one of the standard models for the study of neurotoxicity. cGMP/PKG, PI13/Akt, and Bcl-2/Bax pathways have been shown to involve in the serum-deprivation-induced toxicity [38]. *M. pruriens* seed extract presented the potential of neuroprotective activity and thus may have a positive impact on aging and neurodegenerative diseases to retard the accelerated rate of neuronal degeneration.

*M. pruriens* seed extract contained substances including 5-hydroxytryptamine, alanine, arachidic acid, arginine, aspartic acid, behenic acid, beta-carboline, beta-sitosterol, bufotenine, choline, cis-12,13-epoxyoctadec-trans-9-cis-acid, cis-12,13-epoxyoctadec-trans-9-enoic-acid, cystine, gallic-acid, glutamic acid, glutathione, glycine, histidine, L-dopa, lecithin, leucine, linoleic acid, n, n-dimethyltryptamine, n, n-dimethyltryptamine-n-oxide, nicotine, phenyalanine, phosphorus, proline, protein, prurienidine, prurienine, saponins, serine, serotonin, threonine, tryptamine, tyrosine, valine, and vernolic acid, according to Dr. Duke’s Phytochemical and Ethnobotanical Databases at Phytochemical Database, USDA-ARS-NGRL, Beltsville Agricultural Research Center [39]. Some of these compounds were AChE inhibitors, for example, physostigmine, which can enhance central levels of synaptic choline [40]. The aromatic amino acids such as tyrosine, phenylalanine, and tryptophan were identified as AChE inhibitors [41]. Pan et al. reported that flavonoid glycoside can inhibit AChE leading to a significant improvement in dyskinesia recovery rate in zebrafish [11]. Cognitive impairment and dementia are common complications of Parkinson’s disease. Patients with Parkinson’s disease with dementia often have significant cholinergic defects, which may be treated with cholinesterase inhibitors [42]. Several reports revealed that the use of an acetylcholinesterase inhibitor might improve cognitive function and reduce the risk of falls in patients with Parkinson’s disease [43]. Therefore, *M. pruriens* seed extract with acetylcholinesterase inhibitory activity may be considered a prospective natural product for the treatment of Parkinson’s disease, which may improve cognitive and motor functions of patients. This study reported the neuroprotective effect and anticholinesterase inhibition of *M. pruriens* seed aqueous extract, which contained large amounts of L-dopa (7%) along with other bioactive compounds including phenolic compounds and flavonoids. *M. pruriens* seed extract obtained in this study suggested more effectiveness and less toxicity than synthetic L-dopa for the treatment of Parkinson’s disease. Our results were supported by Katzenschlager et al. showing the rapid onset of action and a long time without a concomitant increase in dyskinesias on *M. pruriens* seed powder formulation compared with L-dopa preparations in the long-term management of Parkinson’s disease [14]. However, additional research is necessary to determine the agents responsible for other in vivo activities as well as the molecular mechanisms involved in their effects.

## 5. Conclusions

Long-term use of synthetic L-dopa can cause serious side effects such as dyskinesia and abnormal involuntary movement. A search for more effective treatment with lower side effects is significant for improving the quality of life of Parkinson’s patients taking this drug. In this study, we reported the dual effects of *M. pruriens* seed extract on neuroprotective effect and acetylcholinesterase inhibitory activity at specific doses. Our findings suggested that *M. pruriens* seed extract is an attractive candidate for neuroprotection and preventing dyskinesia induced by cholinergic neuronal excitability in dopaminergic-depleted striatum.
